# High Electrode Impedance Values in Pediatric Cochlear Implant Recipients May Imply Insufficient Auditory and Language Skills Development

**DOI:** 10.3390/jcm9020506

**Published:** 2020-02-13

**Authors:** Faris F. Brkic, Sekib Umihanic, Alen Harcinovic, Lejla Piric, Fuad Brkic

**Affiliations:** 1 Department of Otorhinolaryngology, Head and Neck Surgery, Medical University of Vienna, 1090 Vienna, Austria; 2 Department of Otorhinolaryngology, University Clinical Center Tuzla, 75000 Tuzla, Bosnia and Herzegovina; sekib.umihanic@ukctuzla.ba (S.U.); alen.harcinovic@ukctuzla.ba (A.H.); lejla.piric@ukctuzla.ba (L.P.); fuad.brkic@ukctuzla.ba (F.B.)

**Keywords:** electrode impedance values, pediatric cochlear implant, auditory and language skills development

## Abstract

Background: Measurements of electrode impedance values are routinely performed after cochlear implantation. The primary objective of the study was to determine if pediatric, prelingually deafened patients with different postoperative performances showed significantly different impedance values one year after implantation. Methods: This study comprised 42 pediatric cochlear implant recipients provided with the device in a single academic tertiary referral center between 1 January 2000, and 31 December 2016. Medical chart analysis was performed in order to assess evolution of impedance values during the first postoperative year on a monthly basis. Electrode impedance values measurements one year postoperatively were compared between children with successful and unsuccessful auditory and language skills development assessed using the EARS protocol (a name of a performance test). Furthermore, values were compared among recipients of different implant types and among different cochlear segments. Results: A gradual rise of average impedance values was found during the first months of implant use (1st month, 7.32 kΩ; 3rd month, 7.86 kΩ) with the peak at the 4th postoperative month (7.96 kΩ), followed by a gradual decrease towards the 12th month (6th month, 7.62 kΩ; 12th month, 6.86 kΩ). Lower values at the 12th postoperative month were observed in recipients with successful development compared to patients presented with unsuccessful development (6.22 kΩ vs. 7.82 kΩ; *p* = 0.001). Mean impedance values were different when compared among cochlear segments and among different implant types. Conclusion: High electrode impedance values one year after implantation in pediatric patients may imply insufficient auditory and language skills development. Further studies are needed in order to validate our results.

## 1. Introduction

One important utilization of cochlear implants (CI) is a restoration of auditory perception in prelingually deafened children using electrical stimulation of the cochlea. Outcomes of cochlear implantation in prelingually deafened patients can be assessed as speech perception development. Generally, speech perception is a function that depends not only on CI functioning properly, but on other preoperative and postoperative factors as well (duration and cause of hearing loss, age, therapy after implantation, etc.) [1].

Measurements of the electrode impedance (EI) values are performed routinely after cochlear implantation, and in most centers on a monthly basis. These measurements allow assessment of electrode function intraoperatively as well as postoperatively, as well as the monitoring of the implant function during the rehabilitation period [2]. Using these measurements, physicians can effectively evaluate the working status of the device [3]. Moreover, it has been shown that proper fitting of the processor is essential for providing good quality speech perception [4].

Auditory and linguistic performance of children implanted with a CI can be assessed using the EARS protocol (a name of a performance test), which includes the Listening Progress Profile (LiP) and the Monosyllabic-Trochee-Polysyllabic-Word Test (MTP) [5].

The LiP is utilized as a measure of the auditory reaction, identification and discrimination of various sounds. Patients are exposed to different acoustic stimuli. On some sounds, the child is observed whether it is able to differentiate their basic properties (long–short, loud–soft), or whether it is able to identify them. For each item, the examiner rates the child’s responses on a three-point scale and calculates the total score. Assessment with the LiP is recommended for children older than one year of age.

The MTP requires the patient to identify a heard word, and it assesses the auditory and basic linguistic skills of children. Based on individual development, testing is performed with groups of 3 to 12 words with a different number of syllables. With this test, recognition of stress patterns (number of syllables) as well as a child’s ability to understand simple words in a closed-set situation is assessed. It should not be used for patients under the age of two [5].

To the best of our knowledge, this is the first study assessing the impact of EI values on the success of postoperative auditory and language skills development in pediatric, prelingually deafened CI recipients. Furthermore, the impedance values were compared among patients implanted with different implant types, as well as among different cochlear segments. 

## 2. Material and Methods

This retrospective study comprised 42 children provided with a CI, all from one manufacturer (Medel, Innsbruck, Austria) at a single tertiary academic medical referral center (Department of Otorhinolaryngology, University Clinical Center Tuzla, Bosnia and Herzegovina) between 1 January 2000, and 31 December 2016. The mean age of patients at the time of implantation was 50.1 months (range, 21.0 months–8.2 years). Twenty-six (61.9%) patients were male. All patients were prelingually deafened.

Our study protocol included retrospective data including electrode impedance measurements one year after implantation on a monthly basis. Moreover, the data on implant type (Combi 40+, Pulsar Ci100 and Sonata Ti100) and on impedance values in three cochlear segments (basal, medial and apical) were included as well. The EI values after 12 months were compared among children with different postoperative performances. Furthermore, investigated values were compared among three cochlear segments (basal, middle and apical) and among three CI types (Combi 40+, Pulsar Ci100 and Sonata Ti100). Medical chart analysis was performed using the hospital’s database system in order to extract the above mentioned values and the patients’ demographics including age, sex, date of implantation, cortisone use (intratympanic or intravenous) and the surgical technique.

Postoperative performance of CI recipients was analyzed using the EARS Protocol developed by MED-EL, one year postoperatively. This evaluation protocol employed the LiP and the MTP in order to assess postoperative development of implanted children. Based on a combination of results of these two tests, the overall score of 1–4 was given in our center. Auditory and language skills development 12 months after implantation with grades 1 and 2 were rated “successful”, and “unsuccessful” for grades 3 and 4. Assessment of the development of all implanted children was performed by the same speech pathologist.

Statistical analysis was performed using the SPSS 22.0 Software Kit (SPSS Inc., Chicago, IL, USA). The Mann–Whitney U test was used to compare mean EI values between patients with successful and unsuccessful development. The Kruskal–Wallis test was utilized to compare average impedance values among three cochlear segments and three implant types. Significance level (alpha) was set at 0.05 (two tailed test). 

This study was approved by the institutional ethics committee on 22 January 2018 (Number of approval: 02-09/2-1/18).

## 3. Results

Elevations of electrode impedance average values were found during the first months of implant use (1st month, 7.32 kΩ; 3rd month, 7.86 kΩ), were highest at the 4th month after activation (7.96 kΩ), after which the values gradually decreased (6th month, 7.62 kΩ; 12th month, 6.86 kΩ). Average EI values of apical (8.04 kΩ) and middle cochlear segments (6.99 kΩ) were higher than basal segment values (6.95 kΩ), and values of basal and middle cochlear segments decreased from the 1st to the 12th month (basal, 8.4 kΩ–7.13 kΩ; medial, 6.99 kΩ–5.92 kΩ), but values for the apical segment of the cochlea rose (6.95 kΩ–7.53 kΩ). 

**Figure 1:**
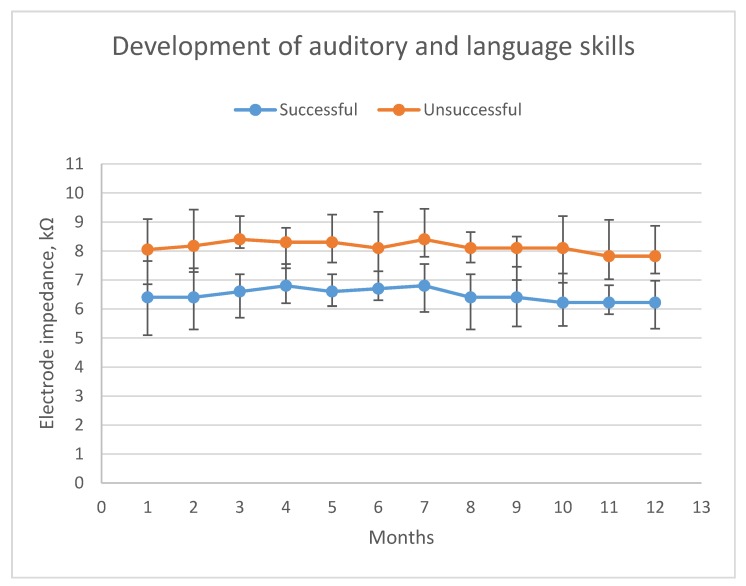
Evolution of EI values in the first 12 postoperative months compared between patients with successful and patients with unsuccessful auditory and language skills development. X axis: time after cochlear implantation in months. Y axis: Values of electrode impedance in kΩ. Blue line: patients with successful development. Red line: patients with unsuccessful development.

**Figure 2:**
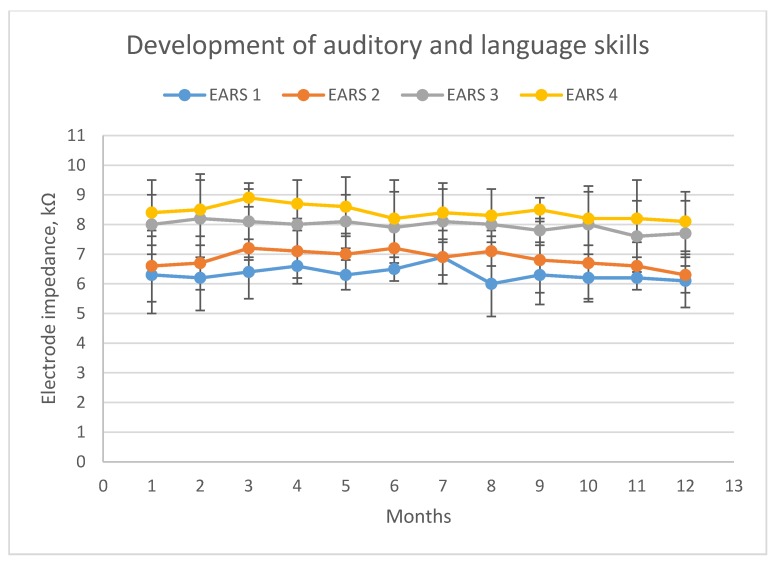
Evolution of EIVs in the first 12 months after activation compared between patients with different EARS scores. X axis: time after cochlear implantation in months. Y axis: Values of electrode impedance in kΩ. Blue line: patients with EARS Score 1. Red line: patients with EARS score 2. Grey line: patients with development score 3. Yellow line: patients with development score 4.

A rehabilitation success assessment one year postoperatively showed successful auditory and language skills development in 25 children (59.5%) (grade 1: 13 patients; grade 2: 12 patients), and unsuccessful development in 17 children (40.5%) (grade 3: 11 patients; grade 4: 6 patients). Patient demographics according to development success are presented in Table 1. Children with successful development had significantly lower EI values after the 12th postoperative month than patients presented with unsuccessful development (6.22 kΩ vs. 7.82 kΩ, respectively; Mann–Whitney U test: *p* = 0.001). Evolution of impedance values for these two patient groups is shown in Figure 1. Furthermore, Figure 2 depicts EI values evolution for patients with different development scores. 

Twenty-seven (64.3%) children were provided with the Combi 40+ implant (mean age, 58.7 months; range: 29.0 months–8.2 years), seven (16.7%) children with the Pulsar Ci100 (mean age, 34.3 months; range, 21 months–4.4 years) and eight (19%) children with the Sonata Ti100 implant (mean age, 37.3 months; range, 21 months–4.6 years). Mean EI values at measurement after 12 months were significantly different in children using different implant types (Combi 40+: 6.23 kΩ, Pulsar Ci100: 7.11 kΩ, Sonata Ti100: 7.54 kΩ; Kruskal–Wallis test: *p* = 0.042). Average impedance values one year postoperatively were not statistically different between male and female patients (6.75 kΩ vs. 7.03 kΩ, respectively; Mann–Whitney U test: *p* = 0.784) and did not correlate with the age of patients at the time of implantation (Pearson Correlation: *p* = 0.341).

All implantations were performed via conventional cochleostomy. Intratympanic or intravenous cortisone was not applied perioperatively in any of the surgeries.

## 4. Discussion

The current study assessed the evolution of electrode impedance in pediatric, prelingually deafened CI recipients and the effect of impedance values 12 months postoperatively on the auditory and language skills development.

There are different reports about EI values evolution after cochlear implantation in pediatric patients. Many authors noted that the values gradually decreased from the 1st to the 12th postoperative month. However, some study groups reported that EI values increased after activation, followed by a period of a decrease and stabilization of the values towards the 12th postoperative month [6,7]. One author stated that the impedance values are at the highest point in the first postoperative month [3]. Our results regarding evolution of EI values correspond with the majority of studies.

As shown in three studies, no significantly different EI values were found among different cochlear segments [8,9,10]; however, one study group noted the opposite [11]. They reported that EI values in apical and middle segments decreased between the first and sixth month and stabilized in following measurements. After three months, impedance values of apical and medial cochlear segment were basically stable, while that of the basal segment further increased. Results of our study did not correspond to these findings. Interestingly, values showed a gradual increase in the apical segment in our patient cohort. The higher EI values in the apical segment may be attributed to the physical characteristics of the Med-El electrode [12]. Furthermore, higher impedance values in the apical segment could be induced by trauma during electrode insertion [13,14].

Electrode impedance values were analyzed for different implant types: Combi 40+ [3,4,11], Nucleus 24M [6,8] and Clarion [15]. In these studies, there were no statistically significant differences in EI values among different CI types. As presented in our study, impedance values were significantly higher in patients implanted with the Sonata Ti100 system than in those provided with the Combi 40+ or the Pulsar.

In order to evaluate the postoperative auditory and language skills development after cochlear implantation, different centers utilize different assessments. Yet, up until now, there is still no widely accepted consensus and standardization in this regard [16]. The newly introduced EARS Protocol (MEDEL Innsbruck, Austria) is recommended for assessment of auditory and language skills development in children [5]. As noted above, it includes the LiP and MTP test. In our center, this evaluation is used for assessment of postoperative performance of pediatric CI recipients. Results of both tests are combined for one of the four final grades: excellent (1), good (2), not satisfactory (3) and bad (4) auditory and language development. The assessment of auditory and linguistic skills development using this testing was performed by the same speech pathologist on all subjects.

To the best of our knowledge, this is the first study analyzing the correlation between the EI values and the development success in children implanted with a CI, and we have been able to show significantly lower impedance values in children with successful auditory and language development.

As noted by one author, surgical technique used for cochlear implantation had an effect on the progression of EI values. This study group showed significantly higher EI values 12 months postoperatively in the conventional cochleostomy group than in the soft surgery group (*p* < 0.05) [17]. In our study, all patients underwent the same operative technique. Moreover, cortisone has proven to lower impedance values after cochlear implantation [18]. Yet, as the use of perioperative steroids in cochlear implantation has not yet been established in our center, all patients were implanted without intratympanic or intravenous cortisone perioperatively.

One of the possible weak points of our study is the limited number of patients (42). However, most of the other studies who have dealt with this issue were also performed on very limited number of patients (16 [6], 18 [19], 20 [13], 24 [3], 25 [11] and 50 patients [20]). Nevertheless, statistical significance of our results was achieved. Certainly, studies on larger samples of respondents are welcome.

## 5. Conclusions

We confirmed that lower electrode impedance values after the first postoperative year were associated with better auditory and language skills development in pediatric, prelingually deafened cochlear implant recipients. There is a need for a universal standardized outcome measure and framework for assessment of auditory and speech development after pediatric cochlear implantation. High electrode impedance values one year after implantation in pediatric patients may imply insufficient auditory and speech skills development. Further studies are needed in order to validate our results.

## Figures and Tables

**Table 1:** Patient demographics.

Auditory and Language Development	Mean Age, Years	Male/Female, *n*	Male/Female, %	EI Values after 12 Months, kΩ	*n*, %
Successful	4.1	15/10	60.0/40.0	6.22 ± 0.75	25/59.5
Unsuccessful	4.3	11/6	64.7/35.3	7.82 ± 1.05	17/40.5
Total	4.2	26/16	61.3/38.1	6.68 ± 0.87	42/100
